# Host co-factors of the retrovirus-like transposon Ty1

**DOI:** 10.1186/1759-8753-3-12

**Published:** 2012-08-02

**Authors:** Jenni K Risler, Alison E Kenny, Ryan J Palumbo, Eric R Gamache, M Joan Curcio

**Affiliations:** 1 Laboratory of Molecular Genetics, Wadsworth Center, Albany, NY, 12201, USA; 2 Department of Biomedical Sciences, School of Public Health, University at Albany, Albany, NY, 12201, USA; 3 Current address: Genomics Shared Resource, Fred Hutchinson Cancer Research Center, Seattle, WA, 98109, USA; 4 Current address: Biology Department, Rensselaer Polytechnic Institute, Troy, NY, 12180, USA

**Keywords:** Ty1, LTR-retrotransposon, Saccharomyces cerevisiae, Host factor, Ribosome, Biogenesis

## Abstract

**Background:**

Long-terminal repeat (LTR) retrotransposons have complex modes of mobility involving reverse transcription of their RNA genomes in cytoplasmic virus-like particles (VLPs) and integration of the cDNA copies into the host genome. The limited coding capacity of retrotransposons necessitates an extensive reliance on host co-factors; however, it has been challenging to identify co-factors that are required for endogenous retrotransposon mobility because retrotransposition is such a rare event.

**Results:**

To circumvent the low frequency of Ty1 LTR-retrotransposon mobility in *Saccharomyces cerevisiae*, we used iterative synthetic genetic array (SGA) analysis to isolate host mutations that reduce retrotransposition. Query strains that harbor a chromosomal Ty1*his3AI* reporter element and either the *rtt101Δ* or *med1Δ* mutation, both of which confer a hypertransposition phenotype, were mated to 4,847 haploid ORF deletion strains. Retrotransposition was measured in the double mutant progeny, and a set of 275 ORF deletions that suppress the hypertransposition phenotypes of both *rtt101Δ* and *med1Δ* were identified. The corresponding set of 275 retrotransposition host factors (RHFs) includes 45 previously identified Ty1 or Ty3 co-factors. More than half of the *RHF* genes have statistically robust human homologs (*E* < 1 x 10^-10^). The level of unintegrated Ty1 cDNA in 181 *rhfΔ* single mutants was altered <2-fold, suggesting that the corresponding co-factors stimulate retrotransposition at a step after cDNA synthesis. However, deletion of 43 *RHF* genes, including specific ribosomal protein and ribosome biogenesis genes and RNA degradation, modification and transport genes resulted in low Ty1 cDNA levels. The level of Ty1 Gag but not RNA was reduced in ribosome biogenesis mutants *bud21Δ*, *hcr1Δ*, *loc1Δ*, and *puf6Δ*.

**Conclusion:**

Ty1 retrotransposition is dependent on multiple co-factors acting at different steps in the replication cycle. Human orthologs of these RHFs are potential, or in a few cases, presumptive HIV-1 co-factors in human cells. *RHF* genes whose absence results in decreased Ty1 cDNA include characterized RNA metabolism and modification genes, consistent with their having roles in early steps in retrotransposition such as expression, nuclear export, translation, localization, or packaging of Ty1 RNA. Our results suggest that Bud21, Hcr1, Loc1, and Puf6 promote efficient synthesis or stability of Ty1 Gag.

## Background

Reverse transcription of RNA generates a significant portion of the eukaryotic genome, including retrotransposons, endogenous retroviruses, retrogenes, processed pseudogenes, and other retrosequences [1,2]. The reverse transcriptases that create retrosequences are encoded by retrotransposons. To understand how eukaryotic hosts harness retrotransposons to create adaptive genome rearrangements and novel genes and regulatory sequences, it is essential to identify host factors that are co-opted for retrotransposon mobility and elucidate their mechanism of action.

Three classes of eukaryotic retrotransposons have been described: LTR (long terminal repeat)-retrotransposons, TP (target-primed)-retrotransposons, and Y (tyrosine-recombinase)-retrotransposons [3]. LTR-retrotransposons, which are structurally and functionally related to infectious retroviruses, are the only transposable elements in the nuclear genome of the budding yeast, *Saccharomyces cerevisiae*. Ty1 elements comprise the most abundant, highly expressed and mobile of the LTR-retrotransposon families in the *S. cerevisiae* genome. Ty1 elements consist of direct terminal repeats flanking two overlapping open reading frames, *gag* (*TYA1*) and *pol* (*TYB1*). The Ty1 mRNA, which is transcribed by RNA polymerase II, capped and polyadenylated, is the template for translation of all Ty1 proteins as well as for reverse transcription of the full-length cDNA. Two primary translation products are synthesized: p49-Gag and p199-Gag-Pol, the latter resulting from a programmed ribosomal frameshift from *gag* to *pol*. Ty1 mRNA is encapsulated into cytoplasmic virus-like particles (VLPs) consisting of Ty1 Gag and Gag-Pol. Inside the VLP, Gag is processed to its mature form (p45-Gag), while Gag-Pol is processed into p45-Gag, protease (PR), integrase (IN), and reverse transcriptase/RNaseH (RT/RH). In mature VLPs, Ty1 RNA is reverse-transcribed into a linear, double-stranded cDNA. The cDNA, in association with IN, is then transported back to the nucleus, where it is integrated into chromosomal DNA [4,5]. Alternatively, Ty1 cDNA can enter the genome by recombination at chromosome break sites [6].

Although the majority of the 30 to 35 Ty1 elements in the genome of *S. cerevisiae* laboratory strains are functional for retrotransposition, and Ty1 RNA is one of the most abundant mRNAs in the cell, there is only one retrotransposition event per 10,000 cells approximately [7-9]. The low frequency of endogenous Ty1 element mobility presents a significant barrier to performing genetic screens for host co-factors that facilitate retrotransposition. The first genetic screen for Ty1 retrotransposition host factors (RHFs) overcame this barrier by using a plasmid-based Ty1 element expressed from the inducible *GAL1* promoter (pGTy1). This screen identified 99 non-essential *RHF* genes that promote pGTy1*HIS3* retrotransposition [10]. However, pGTy1 expression has been shown to override host-mediated transpositional dormancy and copy number control, and therefore it could mask the hypotransposition phenotype of many Ty1 co-factor mutants [11-13]. A recent screen employed an integrating plasmid-based Ty1 element expressed from the native promoter and tagged with the retrotransposition indicator gene, *his3AI*. This screen identified 168 non-essential genes as *RHF*s [14]; however, there was little overlap between the sets of candidate RHFs identified in these two screens, and relatively few of these RHFs have been characterized. Two similar screens for co-factors of the distantly related Ty3 LTR-retrotransposon using a low copy number or high copy number pGTy3 element identified 21 and 66 Ty3 co-factors, respectively, including a few that are also necessary for Ty1 retrotransposition [15-17].

Aside from RHFs that are required for Ty1 transcription (reviewed in [4,5]), several RHFs that promote post-transcriptional steps in retrotransposition of endogenous Ty1 elements have been characterized. Dbr1, an intron RNA lariat debranching enzyme, acts at a post-translational step to stimulate Ty1 cDNA accumulation by a thoroughly investigated but elusive mechanism [18-21]. The mRNA decapping complex, Dcp1-Dcp2, the 5′ to 3′ mRNA exonuclease, Xrn1, and components of the deadenylation-dependent mRNA decay pathway (Dhh1, Lsm1, Pat1, and Ccr4) and the nonsense-mediated mRNA decay pathway (Upf1, Upf2, and Upf3) stimulate post-translational steps in retrotransposition [22-24]. The 5′ to 3′ mRNA decay pathways are thought to regulate degradation of a Ty1 antisense transcript that interferes with transposition and to facilitate packaging of Ty1 RNA into VLPs [12,23,24]. Bud22 is a ribosome biogenesis factor required for 40 S ribosomal subunit formation. In a *bud22Δ* mutant, the levels of Ty1 Gag, especially the processed p45-Gag, and VLPs are decreased, and translational frameshifting from *gag* to *pol* is reduced [14]. Hos2 and Set3, components of the SET3 histone deacetylase complex, promote integration of Ty1 cDNA [25].

The goal of this study was to identify a more complete set of RHFs that promote retromobility of endogenous chromosomal Ty1 elements. A chromosomal Ty1 element marked with *his3AI* gives rise to marked Ty1*HIS3* retrotransposition events in one in approximately 10^7^ cells [7]. To identify host co-factors that are necessary for these rare events, we used an iterative synthetic genetic array (SGA) approach. This method involved screening the non-essential ORF deletion collection for gene deletions that suppress the hypertransposition phenotypes of two different mutants. One of the hypertransposition mutants carried a deletion of *RTT101*, a gene encoding the cullin-component of an E3 ubiquitin ligase. Rtt101 functions in DNA replication fork protection and non-functional rRNA decay [26]. The second was a deletion of *MED1*, which encodes a non-essential subunit of the RNA polymerase II mediator complex involved in transcriptional regulation [27]. Ty1 retrotransposition and cDNA are increased post-transcriptionally in both *rtt101Δ* and *med1Δ* mutants, but by different mechanisms [28,29]. The DNA damage checkpoint pathway is essential for the hypertransposition phenotype of an *rtt101Δ* mutant, whereas deletion of genes encoding components of the DNA damage checkpoint pathway has no effect on hypertransposition in a *med1Δ* mutant. Because the hypertransposition phenotypes result from perturbation of distinct pathways, we reasoned that genes whose deletion suppresses hypertransposition in both *rtt101Δ* and *med1Δ* mutants would encode general activators of retrotransposition. Here we describe the identification of 275 candidate Ty1 RHFs. Forty-five were previously identified as Ty1 or Ty3 co-factors in small or high-throughput genetic screens, providing verification of the RHFs identified by the iterative SGA approach. Moreover, 43 *rhfΔ* mutations result in low Ty1 cDNA levels in the absence of either query mutation, indicating that the corresponding RHFs function during or prior to cDNA accumulation. Genes involved in ribosome biogenesis were enriched in the entire set of 275 RHFs and in the subset with reduced cDNA. We provide evidence that ribosome biogenesis factors, Bud21, Hcr1, Loc1, and Puf6 are required for efficient Gag protein synthesis or stability.

## Results

### Iterative synthetic genetic array screen for *RHF* genes

**Figure 1:**
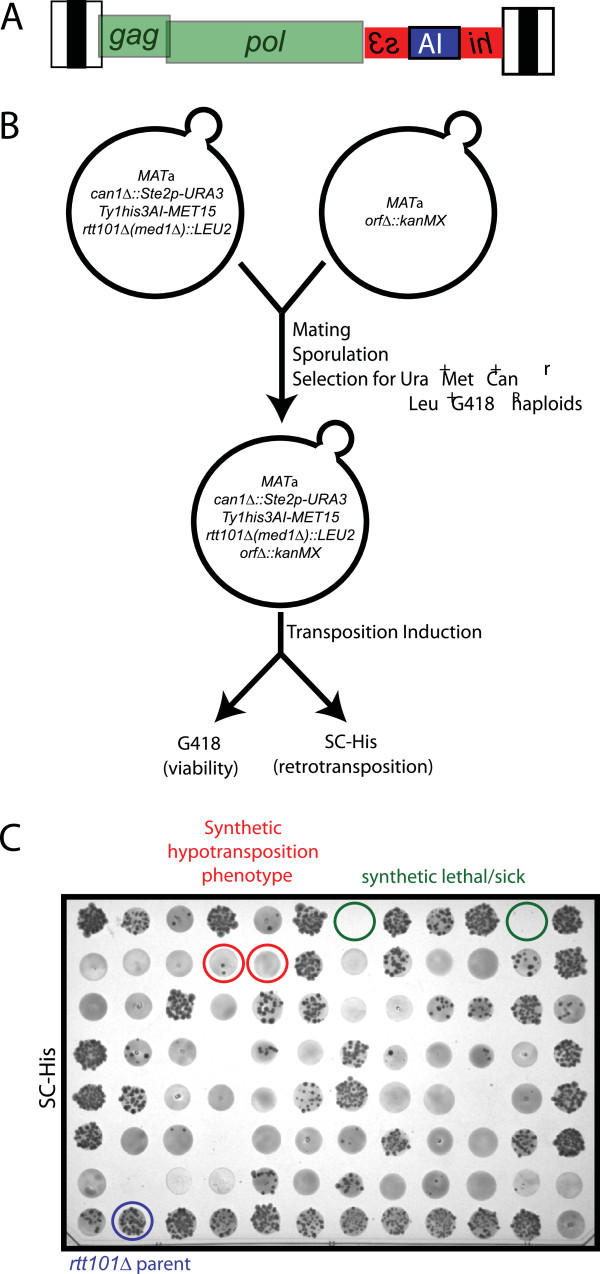
**Modified synthetic genetic array screen for *rhfΔ *mutants.** (**A**) Schematic of the Ty1*his3AI* element that is used to assay the frequency of retrotransposition. Ty1 long terminal repeats are represented as tripartite black and white boxes that flank internal region of the element. The internal region has two partially overlapping ORFs, *gag* and *pol* (green rectangles). As illustrated, the *gag* ORF begins in the 5′ LTR. The *his3AI* retrotransposition indicator gene is inserted in non-coding DNA between the end of *pol* and the beginning of the 3′ LTR. The *his3AI* gene consists of a *HIS3* gene (red rectangles) in the opposite orientation to *gag* and *pol. HIS3* is interrupted by insertion of an artificial intron (AI; blue rectangle) in an unspliceable orientation; however, AI is spliced from the Ty1*his3AI* transcript. When splicing occurs and the transcript is reverse transcribed, a Ty1*HIS3* cDNA is produced, which, when integrated into the genome, confers a His^+^ phenotype. (**B**) Schematic of the genetic manipulations used to generate haploid progeny containing the Ty1*his3AI* element, the *rtt101Δ* or *med1Δ* mutation, and an *orfΔ* mutation. The query strain containing the *rtt101Δ* or *med1Δ* and the *Ty1his3AI-MET15* allele was mated to each *orfΔ::kanMX* strain in the yeast ORF deletion library. Following induction of transposition by growth of cells in YEPD broth at 20°, progeny were plated on YEPD agar containing G418 to assess growth (not shown) and SC -His agar to measure retrotransposition. (**C**) The results of the Ty1*his3AI* retrotransposition assay on one SC -His plate of *rtt101Δ: LEU2orfΔ:kanMX* progeny. Cells that sustained a retrotransposition event give rise to His^+^ papillae, which were counted at each address. Addresses that are blank lack progeny because of synthetic lethality or slow growth (green circles). Addresses with ≤5 His^+^ papillae (red circles) harbor progeny with reduced retrotransposition. The parental *rtt101Δ* strain (blue circle) was plated in an empty address prior to induction of retrotransposition.

To identify co-factors required for Ty1 retrotransposition, we designed a genetic screen using a modification of the SGA protocol [30,31]. First, we constructed a strain carrying a single chromosomal Ty1*his3AI* element adjacent to a selectable marker (Figure 1A). Insertion of the retrotransposition indicator gene *his3AI* into a chromosomal Ty1 element allows cells in which this marked element undergoes retrotransposition to be detected as His^+^ prototrophs [7]. Strain Y9230, which carries a *can1Δ*::*Ste2p-URA3* allele for selection of haploid *MAT*a progeny [31], was modified by introducing *his3AI* into the 3′ untranslated region of *YJRWTy1-2*, and the *MET15* marker downstream of *YJRWTy1-2*. Subsequently, the *rtt101Δ::LEU2* or *med1Δ::LEU2* mutation was introduced into the strain to generate two query strains with elevated levels of Ty1 retromobility. Each query strain was mated to the constituents of the haploid non-essential ORF deletion library (Figure 1B). Diploid strains were sporulated, and aliquots of the spore cultures transferred to a series of selective media plates to obtain haploid *MAT*a progeny that contained the query deletion (*rtt101Δ* or *med1Δ*), the Ty1*his3AIMET15* allele, and an *orfΔ::KanMX* allele. Haploid progeny of each query strain were subjected to a quantitative assay for Ty1*his3AI* retrotransposition. The haploid strains were grown in YPD broth at 20°, a temperature that is permissive for retrotransposition. An aliquot of each culture was spotted onto YPD agar containing G418 and onto SC-His agar. At each address where haploid progeny grew as a confluent patch on YPD agar with G418, the number of His^+^ papillae was determined as a measure of the frequency of Ty1*his3AI* retrotransposition (Figure 1C).

To ascertain whether our selection protocol yielded progeny that were haploid, we tested 78 Leu^+^ Ura^+^ Met^+^ Can^r^ G418^R^ progeny strains derived from the *rtt101Δ* query strain for sensitivity to 0.05% methylmethanesulfonate (MMS), which is conferred by the recessive *rtt101Δ* mutation. All 78 strains were MMS^S^ (data not shown), indicating that they were haploid.

**Figure 2:**
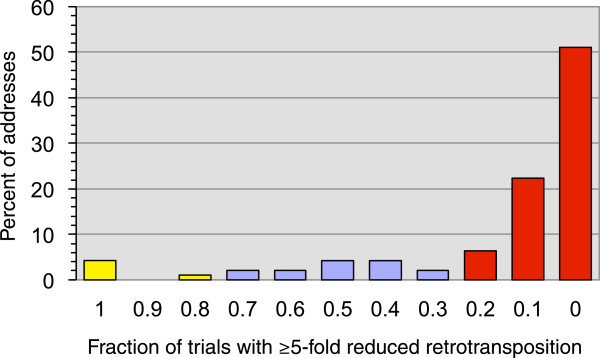
**Reproducibility of results of Ty1 *his3AI *retrotransposition assay across 10 trials.** Progeny of 94 *orfΔ::kanMX* strains on one plate and the *rtt101Δ* query strain were isolated 10 independent times, and retrotransposition was measured in all 940 isolates. The fraction of trials at each address that yielded a ≥5-fold reduction in His^+^ papillae formation relative to the *rtt101Δ* query strain is plotted on the *x*-axis. The percentage of addresses within each category is plotted on the *y*-axis.

A pilot experiment was performed to determine whether the retrotransposition phenotype of progeny strains obtained by SGA selection was reproducible. One plate of 94 yeast *orfΔ::kanMX* strains was mated to the *rtt101Δ* query strain, sporulation was induced, and independent haploid progeny were selected 10 times. All 94 *rtt101Δ orfΔ::kanMX* progeny strains were viable in all 10 trials. The parental *rtt101Δ* strain, which was grown in an empty address on each of the 10 plates of progeny, yielded an average of 25 ± 3 His^+^ papillae per trial. Each trial with haploid progeny at each address was assigned to a binary class depending on whether or not there was a ≥ 5-fold reduction in His^+^ papillae relative to the average for the *rtt101Δ* strain. We determined the fraction of trials at each address that fell into the ≥5-fold reduced retrotransposition category or <5-fold reduced category (Figure 2). At 84 of the 94 (89%) addresses, retrotransposition was reduced ≥5-fold in eight or more of the 10 trials (Figure 2, yellow bars) or in two to zero trials (Figure 2, red bars). Only 10 of the 94 addresses had fewer than eight trials in one category or the other (Figure 2, blue bars). Thus, the results of the retrotransposition assay in independently derived progeny of the same genotype were highly reproducible.

The protocol was applied genome-wide by mating *rtt101Δ* and *med1Δ* query strains to 4,847 haploid ORF deletion strains. Following sporulation, independent haploid progeny were selected twice from spores derived from each query strain. Both sets of progeny from each query strain were tested to determine the retrotransposition frequency. When mated to the *rtt101Δ* query strain, 3,797 ORF deletion strains yielded viable haploid progeny in both trials. Of these, 1,419 strains had ≤5 His^+^ papillae in each trial. Since the parental *rtt101Δ* query strain tested in parallel on each plate yielded an average of 24.4 ± 0.6 His^+^ papillae, ≤5 His^+^ papillae represents a ≥5-fold reduction in retrotransposition. Using the *med1Δ* query strain, 4,289 of the ORF deletion strains yielded viable progeny in both trials. The parental *med1Δ* query strain had an average of 14.0 ± 0.6 His^+^ papillae, and 820 haploid progeny strains had ≤3 His^+^ papillae in each trial, representing a ≥5-fold reduction in retrotransposition. The set of 1,419 gene deletions that reduced Ty1*his3AI* retrotransposition ≥5-fold in an *rtt101Δ* background and the set of 820 gene deletions that reduced retrotransposition ≥5-fold in a *med1Δ* background included 279 gene deletions that were common to both sets (see Additional file 1). Four of the corresponding genes are required for histidine biosynthesis; therefore, the retrotransposition assay was not functional in these strains. The remaining 275 genes encode putative retrotransposition co-factors. Since 3,448 ORF deletion strains yielded viable haploid progeny in all four trials, the probability that 275 *rhfΔ* gene deletions would be present in the intersecting set by chance is low (*P* =6.84 x 10^-8^).

### 275 RHFs identified in overlapping screen sets

Of the 275 RHFs identified by SGA analysis, 45 (16%) were identified previously as Ty1 or Ty3 retrotransposition co-factors (Table 1[10,14-16]). Of these, 26 of were found in a screen for activators of an integrating plasmid-based Ty1*his3AI* element [14]. The fact that single mutants lacking these 45 co-factors are defective for retromobility of plasmid-based Ty1 or Ty3 elements provides confirmation that the modified SGA screen successfully identified *bona fide* Ty1 co-factors.

**Table 1:** RHFs identified as Ty1 or Ty3 RHFs in earlier genetic screens

**RHF**	**Systematic gene name**	**Cellular function**	**Reference**	**Relative Ty1 cDNA level (*rhfΔ/RHF*)**
Apq12	YIL040W	Protein required for nuclear envelope morphology, nuclear pore complex localization, mRNA export from the nucleus; exhibits synthetic lethal genetic interactions with genes involved in lipid metabolism	[14]	1.39
Bro1	YPL084W	Class E vacuolar protein sorting factor that coordinates deubiquitination in the multivesicular body (MVB) pathway	[16]	1.54
Ccr4	YAL021C	Component of the CCR4-NOT transcriptional complex, which is involved in regulation of gene expression; component of the major cytoplasmic deadenylase, which is involved in mRNA poly(A) tail shortening	[23]	0.65
Cdc50	YCR094W	Endosomal protein that interacts with phospholipid flippase Drs2p; interaction with Cdc50p is essential for Drs2p catalytic activity	[14]	0.38
Cpr7	YJR032W	Peptidyl-prolyl cis-trans isomerase, Hsp90 co-chaperone	[10,16]	0.67
Dhh1	YDL160C	Cytoplasmic DExD/H-box helicase, stimulates mRNA decapping	[14,16]	0.23
Elp2	YGR200C	Subunit of transcriptional elongator complex (HAT)	[10]	0.21
Glo2	YDR272W	Cytoplasmic glyoxalase II, catalyzes the hydrolysis of S-D-lactoylglutathione into glutathione and D-lactate	[14]	1.04
Hda3	YPR179C	Subunit of a possibly tetrameric trichostatin A-sensitive class II histone deacetylase complex that contains an Hda1p homodimer and an Hda2p-Hda3p heterodimer; required for the activity of the complex	[14]	1.84
Hmo1	YDR174W	Chromatin associated high mobility group (HMG) family member involved in genome maintenance; rDNA-binding component of the Pol I transcription system; associates with a 5′-3′ DNA helicase and Fpr1p, a prolyl isomerase	[14]	0.19
Ksp1	YHR082C	Nuclear serine/threonine kinase; stress response	[15]	1.79
Loc1	YFR001W	Nuclear protein involved in asymmetric localization of ASH1 mRNA; binds double-stranded RNA *in vitro*; constituent of 66 S pre-ribosomal particles	[14]	0.14
Lsm1	YJL124C	Component of heteroheptameric complex involved in cytoplasmic mRNA degradation	[10,14]	0.53
Met5	YJR137C	Sulfite reductase, involved in amino acid biosynthesis and transcription repressed by methionine	[15]	1.04
Mig3	YER028C	Probable transcriptional repressor involved in response to toxic agents that inhibit ribonucleotide reductase; phosphorylation by Snf1p or the Mec1p pathway inactivates Mig3p, allowing induction of damage response genes	[14]	1.75
Ncl1	YBL024W	tRNA:m5C-methyltransferase	[15]	0.49
Nip100	YPL174C	Large subunit of the dynactin complex, which is involved in partitioning the mitotic spindle between mother and daughter cells; putative ortholog of mammalian p150 (glued)	[14]	1.06
Nup133	YKR082W	Subunit of the Nup84p subcomplex of the nuclear pore complex	[10]	3.13
Nup170	YBL079W	Subunit of the nuclear pore complex (NPC), required for NPC localization of specific nucleoporins; involved in nuclear envelope permeability and chromosome segregation; has similar to Nup157; essential role, with Nup157, in NPC assembly	[14]	0.66
Oca4	YCR095C	Cytoplasmic protein required for replication of Brome mosaic virus in *S. cerevisiae*	[14]	0.43
Pde2	YOR360C	High-affinity cyclic AMP phosphodiesterase, component of the cAMP-dependent protein kinase signaling system, protects the cell from extracellular cAMP	[14]	0.92
Ref2	YDR195W	RNA-binding protein involved in the cleavage step of mRNA 3′-end formation prior to polyadenylation, and in snoRNA maturation; part of holo-CPF subcomplex APT, which associates with 3′-ends of snoRNA- and mRNA-encoding genes	[14]	0.17
Rpl16B	YNL069C	Component of the large (60 S) ribosomal subunit; binds to 5.8 S rRNA; has similarity to Rpl16A	[10]	0.51
Rpl27a	YHR010W	Protein component of the large (60 S) ribosomal subunit, nearly identical to Rpl27B	[14]	0.19
Rpp1A	YDL081C	Ribosomal stalk protein P1 alpha, involved in the interaction between translational elongation factors and the ribosome	[10]	1.23
Rps19b	YNL302C	Protein component of the small (40 S) ribosomal subunit, required for assembly and maturation of pre-40 S particles; mutations in human RPS19 are associated with Diamond Blackfan anemia; nearly identical to Rps19A	[14]	0.28
Rps25a	YGR027C	Protein component of the small (40 S) ribosomal subunit; nearly identical to Rps25B	[14]	0.19
Ski8	YGL213C	Ski complex component and WD-repeat protein, mediates 3′-5′ RNA degradation by the cytoplasmic exosome; also required for meiotic double-strand break recombination	[14]	0.58
Snf5	YBR289W	Subunit of the SWI/SNF chromatin remodeling complex involved in transcriptional regulation; functions interdependently in transcriptional activation with Snf2p and Snf6p	[32]	0.09
Snf6	YHL025W	Subunit of the SWI/SNF chromatin remodeling complex involved in transcriptional regulation; functions interdependently in transcriptional activation with Snf2p and Snf5p	[32]	0.27
Spt3	YDR392W	Subunit of the SAGA and SAGA-like transcriptional regulatory complexes, interacts with Spt15p to activate transcription of some RNA polymerase II-dependent genes	[33]	0.09
Spt8	YLR055C	Subunit of the SAGA transcriptional regulatory complex	[14]	0.10
Spt10	YJL127C	Putative histone acetylase; sequence-specific activator of histone genes	[10,14]	1.17
Sqs1	YNL224C	Stimulates the ATPase and helicase activities of Prp43p; acts with Prp43p to stimulate 18 s rRNA maturation by Nob1p; component of pre-ribosomal particles	[14]	0.63
Sse1	YPL106C	ATPase; Hsp90 co-chaperone; binds unfolded proteins; member of the heat shock protein 70 (HSP70) family	[10]	0.25
Swi3	YJL176C	Subunit of the SWI/SNF chromatin remodeling complex	[14]	0.44
Tgs1	YPL157W	Trimethyl guanosine synthase, conserved nucleolar methyl transferase that converts the m(7)G cap structure of snRNAs, snoRNAs, and telomerase *TLC1* RNA to m(2,2,7)G; also required for ribosome synthesis and nucleolar morphology	[14]	1.44
Thp2	YHR167W	Subunit of the THO/TREX complex, couples transcription to mRNA export	[10]	0.98
Trk1	YJL129C	Component of the Trk1p-Trk2p high-affinity potassium transport system; plasma membrane protein	[10,14]	1.14
Ump1	YBR173C	Chaperone required for correct maturation of the 20 S proteasome	[14]	0.36
Upf1	YMR080C	ATP-dependent RNA helicase of the SFI superfamily involved in nonsense-mediated mRNA decay (NMD); required for efficient translation termination at nonsense codons and targeting of NMD substrates to P-bodies; involved in telomere maintenance	[14,23]	0.25
Upf3	YGR072W	Component of the NMD pathway, along with Nam7 and Nmd2/Upf2; involved in decay of mRNA containing nonsense codons	[14,23]	0.29
Vma16	YHR026W	Subunit c of vacuolar-ATPase, which functions in acidification of the vacuole; one of three proteolipid subunits of the V^0^ domain	[15]	1.32
Vph1	YOR270C	Subunit a of vacuolar-ATPase, V^0^ domain which functions in acidification of the vacuole; one of three proteolipid subunits of the V^0^ domain	[10,16]	1.55
YML009C-A	YML009C-A	Dubious ORF unlikely to encode a functional protein, based on available experimental and comparative sequence data; ORF overlaps the essential gene, *SPT5*	[14]	N.D.

The 275 candidate *RHF*s include 190 (69%) that have statistically significant human homologs (*E*-value score of <0.01; see Additional file 1), and 149 (54%) that have *E-*value scores of <1 x 10^-10^, suggesting evolutionary and potentially functional conservation. Twenty-one of the 275 *RHF*s are encoded by misidentified or dubious ORFs. Many of these ORFs partially overlap characterized genes, which could play a role in retrotransposition; however, the effects of overlapping ORFs on retrotransposition have not been investigated further.

**Figure 3:**
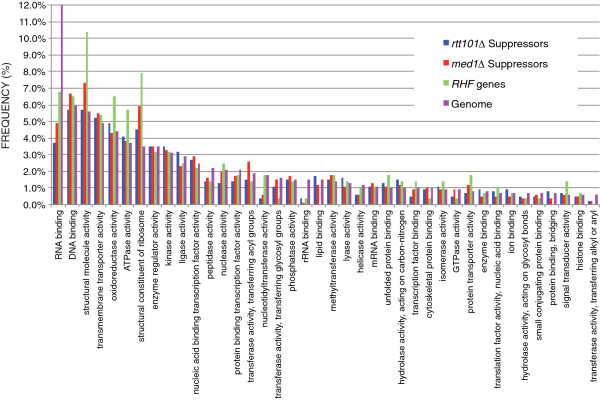
**Frequency of distribution of suppressors of *rtt101Δ *hypertransposition, suppressors of *med1Δ *hypertransposition, *RHF *genes, and all yeast genes in Gene Ontology molecular function categories.** The histogram indicates the percent of the total number of genes in each gene set that are found in each GO molecular function category. The GO categories were assigned using yeast GO slim mapper ( www.yeastgenome.org/cgi-bin/GO/goSlimMapper.pl).

To explore the cellular role of RHFs, we used GO Slim Mapper to assign the *RHF* genes to gene ontology categories based on molecular function and biological process (see additional file 2). A histogram showing the distribution of (a) suppressors of *rtt101Δ* hypertransposition, (b) suppressors of *med1Δ* hypertransposition, and (c) *RHF* genes (that is, genes in the intersecting set of suppressors) compared to the distribution of (d) all yeast genes in GO functional categories is shown in Figure 3. The *rtt101Δ* suppressors and *med1Δ* suppressors were distributed among all GO functional categories and the frequencies of distribution were similar in most categories, which suggests that both screens were biased toward general activators of retrotransposition rather than *rtt101Δ*- or *med1Δ*-specific suppressors. In a small number of categories, notably lipid-binding genes, the frequencies of *rtt101Δ* suppressors and *med1Δ* suppressors were equivalent, but there was little or no overlap between the sets of genes identified, resulting in a low frequency of *RHF* genes in the category. However, *RHF* genes were found in most GO functional categories. In a small number of categories, the frequency of *RHF* genes is substantially higher (for example, structural constituent of ribosome) or lower (for example, rRNA binding) compared to the genome-wide frequency, but most functional categories have similar frequencies of *RHF* genes and all genes. Overall, the data reveal abroad distribution of *RHF* genes among functional gene categories, which is likely to reflect the fact that host factors are required for many steps of Ty1 retrotransposition.

We used FunSpec ( http://funspec.med.utoronto.ca) to determine whether our set of RHFs was significantly enriched for any of 459 MIPS functional categories and found that ribosomal proteins were enriched (*P* = 7.39 × 10^-06^). The screen identified 26 of 246 ribosomal proteins, including the large ribosomal subunit constituents Rpl7a, Rpl16b, Rpl19a, Rpl27a, Rpl31a, Rpl33b, Rpl34a, Rpl37a, and Rpl43a, small ribosomal subunit components Rps11a, Rps19a, Rps19b, Rps25a, Rps27b, and Rps30a, ribosomal stalk protein Rpp1a, ribosome biogenesis factors Rsa3 and Dpb7, translation initiation factor eIF2A (encoded by *YGR054W*), and mitochondrial ribosomal subunits Mrpl7, Mrpl8, Mrpl39, Mrpl49, Mrps28, and Mrp17. The final protein identified was Met13, which is erroneously classified as a mitochondrial ribosomal protein. In addition to ribosomal proteins identified by FunSpec, seven additional ribosome biogenesis factors (Bud21, Hcr1, Loc1, Mrt4, Rkm4, Sqs1, and Utp30) and a ribosome-associated protein chaperone (Zuo1), were identified. Thus, 33 of the 275 RHFs (12%) are constituents of the ribosome or required for ribosome biogenesis.

### Stringency of iterative SGA screen

Deletion strains that did not yield viable progeny in all four trials, or whose progeny did not show a ≥5-fold reduction in Ty1*his3AI* retromobility in all four trials were not identified as *rhfΔ* mutants. Thus, some Ty1 co-factor mutants may not have been found by iterative SGA analysis because of synthetic lethality under transposition-induction conditions or because their absence did not strongly suppress hypertransposition in both the *med1Δ* and the *rtt101Δ* mutants. To understand the limitations of the screen, we examined the results for eight previously characterized Ty1 co-factor genes that were not successfully identified here as *RHF* genes. Seven of eight known Ty1 co-factor mutants were not identified because the mutation failed to suppress retrotransposition in one or both trials of either the *rtt101Δ* screen or the *med1Δ* screen. The co-factor gene deletion *bud22Δ* failed to suppress *rtt101Δ* hypertransposition in either trial, while *tec1Δ* did not suppress *rtt101Δ* hypertransposition in one trial. On the other hand, retrotransposition-defective *xrn1Δ*, *hos2Δ*, *set3Δ*, *pat1Δ*, and *upf2Δ* mutations failed to suppress *med1Δ* hypertransposition in one or both (*hos2Δ*) trials. The eighth Ty1 co-factor mutant, *dbr1Δ*, was not identified because the mutant did not yield viable progeny in one trial with the *rtt101Δ* query strain. In summary, these results suggest that the set of 275 RHFs is not complete, and that the stringency of the SGA screen was a significant limitation to identifying a complete set of non-essential Ty1 co-factors.

### Forty-three RHFs are required for synthesis or stability of Ty1 cDNA

To identify RHFs that act before or during Ty1 cDNA synthesis, we measured the level of unintegrated cDNA produced from endogenous Ty1 elements in *rhfΔ* single mutants. Ty1 cDNA is measured by a Southern blot assay that compares the level of unintegrated Ty1 cDNA to the level of genomic Ty1 element DNA [34]. One to seven biological replicates of 252 of the 275 *rhfΔ* mutants were analyzed. (Ty1 cDNA levels were not determined in strains with deletions of dubious or misidentified ORFs.) Total Ty1 cDNA was reduced to <50% of wild-type levels in 43 of the 275 *rhfΔ* mutants (16%; Table 2). This reduction in cDNA was observed in the absence of either the *rtt101Δ* or *med1Δ* mutation and independently of the Ty1*his3AI* assay. Since Ty1 cDNA is a required intermediate in retrotransposition, these mutants are expected to have lower levels of retrotransposition resulting from the decreased levels of total Ty1 cDNA. Therefore, the results confirm that these 43 *RHF* genes encode host factors that are required for Ty1 retrotransposition. Indeed, eight were previously characterized mutants with defects in Ty1 RNA expression (*swi3Δ, snf5Δ*, *snf6Δ*, *spt3Δ*, and *spt8Δ*) or post-translational steps in retrotransposition (*dhh1Δ*, *upf1Δ*, and *upf3Δ*). A further demonstration that *rhfΔ* mutants with reduced levels of Ty1 cDNA are defective in retrotransposition was obtained by introducing the *elp2Δ* and *dfg10Δ* mutations into a strain containing Ty1*his3AI*. The retrotransposition frequency in *elp2Δ* and *dfg10Δ* mutants was ≤2% and 3.2 (± 2.0)% of the wild-type strain, respectively. Five additional *rhfΔ* mutants with defects in ribosome biogenesis were also shown to have reduced levels of Ty1*his3AI* retrotransposition that are correlated with decreased Ty1 cDNA levels (see below.)

**Table 2:** ***rhfΔ *mutants with > 2-fold reduction in Ty1 cDNA**

**Retrotransposition host factor**	**Systematic ORF name**	**Cellular function**	**Relative Ty1 cDNA level (*rhfΔ*/*RHF*)**	**Genomic DNA samples analyzed (*n*)**
Afr1	YDR085C	Protein required for pheromone-induced projection (shmoo) formation; regulates septin architecture during mating; has an RVXF motif that mediates targeting of Glc7 to mating projections; interacts with Cdc12	0.42	2
Atp17	YDR377W	Subunit f of the F0 sector of mitochondrial F1F0 ATP synthase, which is a large, evolutionarily conserved enzyme complex required for ATP synthesis	0.34	2
Bud21	YOR078W	Also known as UTP16; component of small ribosomal subunit (SSU) processosome that contains U3 snoRNA	0.29	2
Cdc50	YCR094W	Endosomal protein that interacts with phospholipid flippase Drs2; interaction with Cdc50p is essential for Drs2 catalytic activity; mutations affect cell polarity and polarized growth	0.38	2
Cth1	YDR151C	Member of the CCCH zinc finger family; has similarity to mammalian Tis11 protein, which activates transcription and also has a role in mRNA degradation; may function with Tis11 in iron homeostasis	0.30	2
Dbf20	YPR111W	Ser/Thr kinase involved in late nuclear division, one of the mitotic exit network (MEN) proteins; necessary for the execution of cytokinesis; ortholog of human NDR2 kinase	0.45	3
Dbp7	YKR024C	Putative ATP-dependent RNA helicase of the DEAD-box family involved in ribosomal biogenesis	<0.01	2
Dfg10	YIL049W	Probable polyprenol reductase that catalyzes conversion of polyprenol to dolichol, the precursor for N-glycosylation; mutations in human ortholog SRD5A3 confer CDG1Q (Congenital Disorders of Glycosylation type 1Q)	0.27	2
Dgr2	YKL121W	Protein of unknown function; null mutant is resistant to 2-deoxy-D-glucose	0.32	3
Dhh1	YDL160C	Cytoplasmic DExD/H-box helicase, stimulates mRNA decapping, coordinates distinct steps in mRNA function and decay, interacts with both the decapping and deadenylase complexes; ortholog of the human oncogene DDX6/p54/RCK	0.23	5
Elp2	YGR200C	Subunit of elongator complex, which is a component of the RNA polymerase holoenzyme and required for modification of wobble uridines in tRNA; ortholog of human ELP2/STATIP1 gene	0.21	2
Hcr1	YLR192C	Dual function protein involved in translation initiation as a substoichiometric component (eIF3j) of translation initiation factor 3 (eIF3) and required for processing of 20 S pre-rRNA; ortholog of human EIF3J gene	0.29	3
Hit1	YJR055W	Unknown function, required for growth at high temperature	0.24	2
Hmo1	YDR174W	Chromatin associated high mobility group (HMG) family member involved in genome maintenance; rDNA-binding component of the Pol I transcription system; associates with a 5′-3′ DNA helicase and Fpr1, a prolyl isomerase	0.19	3
Kgd1	YIL125W	Component of the mitochondrial alpha-ketoglutarate dehydrogenase complex, which catalyzes a key step in the tricarboxylic acid (TCA) cycle, the oxidative decarboxylation of alpha-ketoglutarate to form succinyl-CoA; ortholog of human OGDHL gene	0.27	2
Loc1	YFR001W	Nuclear protein involved in asymmetric localization of ASH1 mRNA; binds double-stranded RNA *in vitro*; co-localizes with large subunit precursor of ribosome	0.14	3
Los1	YKL205W	Nuclear pore protein involved in nuclear export of pre-tRNA and in re-export of mature tRNAs after retrograde import from the cytoplasm; ortholog of human exportin-T gene, XPOT	0.44	3
Lst7	YGR057C	Protein possibly involved in a post-Golgi secretory pathway; required for the transport of nitrogen-regulated amino acid permease Gap1 from the Golgi to the cell surface	0.44	4
Mrt4	YKL009W	Protein involved in mRNA turnover and large ribosome assembly, co-localizes with large subunit precursor of ribosome; ortholog of human MRTO4 gene	0.17	2
Ncl1	YBL024W	S-adenosyl-L-methionine-dependent tRNA: m5C-methyltransferase, methylates cytosine to m5C at several positions in tRNAs and intron-containing pre-tRNAs; similar to Nop2 and human proliferation associated nucleolar protein p120	0.49	4
Oca4	YCR095C	Cytoplasmic protein required for replication of Brome mosaic virus in *S. cerevisiae*, which is a model system for studying replication of positive-strand RNA viruses	0.43	2
Ref2	YDR195W	RNA-binding protein involved in the cleavage step of mRNA 3′-end formation prior to polyadenylation, and in snoRNA maturation; part of holo-CPF subcomplex APT, which associates with 3′-ends of snoRNA- and mRNA-encoding genes	0.17	2
Rkm4	YDR257C	Ribosomal lysine methyltransferase specific for monomethylation of Rpl42a and Rpl42b (lysine 55); nuclear SET-domain containing protein	0.41	2
Rpl7a	YGL076C	Protein component of the large (60 S) ribosomal subunit, nearly identical to Rpl7b; ortholog of human L7 ribosomal protein gene	0.15	4
Rpl19a	YBR084C-A	Protein component of the large (60 S) ribosomal subunit, nearly identical to Rpl19b; ortholog of human L19 ribosomal protein gene	0.24	2
Rpl27a	YGL076C	Protein component of the large (60 S) ribosomal subunit; nearly identical to Rpl27b; ortholog of human L27 ribosomal protein gene	0.19	2
Rpl31a	YDL075W	Protein component of the large (60 S) ribosomal subunit, nearly identical to Rpl31b; ortholog of human L31 ribosomal protein gene	0.10	2
Rpl43a	YPR043W	Protein component of the large (60 S) ribosomal subunit, identical to Rpl43b; ortholog of human ribosomal protein L37 gene	0.15	3
Rps19b	YNL302C	Protein component of the small (40 S) ribosomal subunit, required for assembly and maturation of pre-40 S particles; mutations in human RPS19 are associated with Diamond Blackfan anemia; nearly identical to Rps19a	0.28	4
Rps25a	YGR027C	Protein component of the small (40 S) ribosomal subunit; nearly identical to Rps25b; ortholog of human S25 ribosomal protein gene	0.19	2
Rps30a	YLR287C-A	Protein component of the small (40 S) ribosomal subunit; nearly identical to Rps30B; ortholog of human S30 ribosomal protein	0.20	2
Snf5	YBR289W	Subunit of the SWI/SNF chromatin remodeling complex involved in transcriptional regulation; functions interdependently in transcriptional activation with Snf2 and Snf6	0.09	2
Snf6	YHL025W	Subunit of the SWI/SNF chromatin remodeling complex involved in transcriptional regulation; functions interdependently in transcriptional activation with Snf2 and Snf5	0.27	2
Snt1	YCR033W	Subunit of the Set3C deacetylase complex that interacts directly with the Set3C subunit, Sif2p; putative DNA-binding protein	0.21	2
Spf1	YEL031W	P-type ATPase, ion transporter of the ER membrane involved in ER function and Ca2+ homeostasis; required for regulating Hmg2 degradation	0.42	2
Spt3	YDR392W	Subunit of the SAGA and SAGA-like transcriptional regulatory complexes, interacts with Spt15 to activate transcription of some RNA polymerase II-dependent genes; also inhibits transcription at some promoters	0.09	3
Spt8	YLR055C	Subunit of the SAGA transcriptional regulatory complex but not present in SAGA-like complex SLIK/SALSA, required for SAGA-mediated inhibition at some promoters	0.10	2
Sse1	YPL106C	ATPase that is a component of the heat shock protein Hsp90 chaperone complex; binds unfolded proteins; member of the HSP70 family	0.25	2
Swi3	YJL176C	Subunit of the SWI/SNF chromatin remodeling complex	0.44	3
Ump1	YBR173C	Short-lived chaperone required for correct maturation of the 20 S proteasome; may inhibit premature dimerization of proteasome half-mers; degraded by proteasome upon completion of its assembly	0.36	3
Upf1	YMR080C	Also known as Nam7; ATP-dependent RNA helicase of the SFI superfamily involved in nonsense mediated mRNA decay; required for efficient translation termination at nonsense codons and targeting of NMD substrates to P-bodies; involved in telomere maintenance	0.25	4
Upf3	YGR072W	Component of the nonsense-mediated mRNA decay (NMD) pathway, along with Upf1 and Upf2; involved in decay of mRNA containing nonsense codons and telomere maintenance; ortholog of human UPF3A and UPF3B genes	0.29	4
YDL124W	YDL124W	NADPH-dependent alpha-keto amide reductase	0.36	2

Unexpectedly, we also identified 29 *RHF* genes whose deletion resulted in a ≥2-fold increase in Ty1 cDNA levels (see Additional file 1). In an earlier study, we found that elevated levels of Ty1 cDNA in two of these *rhfΔ* mutants, *ctf4Δ* and *mms22Δ*, are correlated with increased Ty1 retrotransposition [29]; therefore, these two genes were misidentified as *RHF*s in the SGA analysis. It is not clear why the other 27 *rhfΔ* mutants have increased levels of cDNA. They could also have been misidentified as *rhfΔ* mutants, or perhaps cDNA accumulates in these mutants because of defects in nuclear import or integration of cDNA. For example, the nucleoporin Nup133 was identified here and previously as a pGTy1 co-factor [10], yet deletion causes a >3-fold increase in Ty1 cDNA. Deletion of a second component of the Nup84 complex, Nup120, also increased Ty1 cDNA >3-fold (see Additional file 1).

The remaining 181 *rhfΔ* strains had a <2-fold increase or decrease in Ty1 cDNA levels. The lack of a substantial decrease in cDNA levels in the absence of these RHFs suggests that these putative co-factors promote a late step in retrotransposition. Twenty-three of the *rhfΔ* strains with a <2-fold change in cDNA levels were identified as defective in Ty1 and/or Ty3 retrotransposition in previous screens (Table 1), supporting the idea that these candidate RHFs influence Ty1 retrotransposition even though they do not regulate the level of Ty1 cDNA. As a further test of this concept, we deleted a representative gene, *NAT4*, in a strain carrying a chromosomal Ty1*his3AI* element and measured the effect on retromobility. The retrotransposition frequency in the *nat4Δ* mutant was <3% of that of the congenic wild-type strain, even though the level of Ty1 cDNA in a *nat4Δ* mutant was 101% of that in the wild-type strain. Thus, the histone acetyltransferase Nat4 promotes Ty1 retrotransposition at a step subsequent to Ty1 cDNA accumulation. Together, our results suggest that a large fraction of RHFs influence late steps in retrotransposition.

### Six ribosome biogenesis factors promote a post-transcriptional step in Ty1 retrotransposition

**Figure 4:**
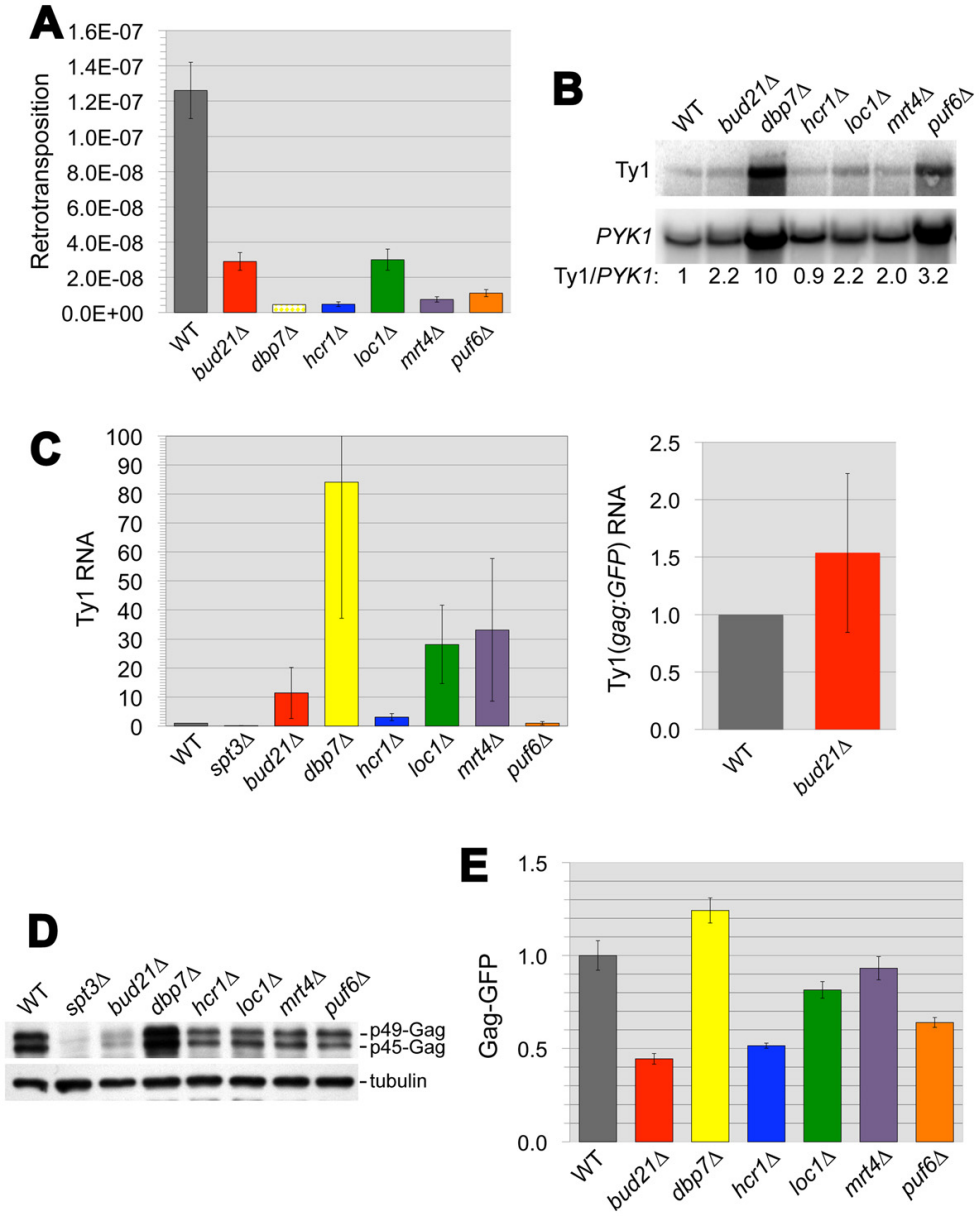
**Levels of Ty1 retromobility, RNA, and Gag: GFP protein in six *rhfΔ *mutants with defects in ribosome biogenesis.** (**A**) The frequency of His^+^ prototroph formation (retrotransposition) in wild-type strain JC3807 (WT) and congenic *rhfΔ* derivatives harboring a chromosomal Ty1*his3AI* element. The frequency reported for the *dbp7Δ* strain is the maximum possible frequency determined as if one His ^+^ colony had formed in each independent culture tested. Error bars: standard error. (**B**) Northern blot analyses of Ty1 RNA (top panel) and *PYK1* RNA (bottom panel) in each strain, using ^32^P-labeled riboprobes. The ratio of ^32^P activity in the Ty1 band to ^32^P activity in the *PYK1* band was determined by phosphorimaging. Ty1/*PYK1* RNA ratios for each strain normalized to that of the wild-type strain are provided below each lane. (**C**) The average level of Ty1 RNA in total RNA from three biological replicates of each strain relative to the wild-type strain was determined by qPCR analysis (left panel). The *spt3Δ* strain is a negative control. The average level of RNA derived from the Ty1(*gag::GFP*)-3566 chromosomal element in total RNA from three biological replicates of the wild type strain and the congenic *bud21Δ* derivative was measured by qPCR analysis. Error bars: standard error. (**D**) Western blot analyses of total cell lysate with anti-VLP antibody, which recognizes unprocessed p49-Gag and processed p45-Gag (top panel), and anti-alpha-tubulin antibody (bottom panel) as a loading control. **(E)** Histogram of the mean Gag:GFP fusion protein activity, as measured by flow cytometry, in *rhfΔ* strains relative to the wild-type strain. Each strain harbors the chromosomal Ty1(*gag::GFP*)-3566 element. Error bars: standard error.

The 43 RHFs that are required for efficient Ty1 cDNA accumulation include eight ribosomal protein paralogs, six ribosome biogenesis factors and a regulator of rRNA transcription (Table 2). Thus, translation of Ty1 RNA could be an important level of host contribution to retrotransposition. We explored the possibility that inefficient Ty1 RNA translation results in retrotransposition and cDNA synthesis defects in ribosome biogenesis factor mutants *bud21Δ*, *dbp7Δ*, *mrt4Δ*, *loc1Δ*, *hcr1Δ*, and *rkm4Δ*. We also analyzed another ribosome biogenesis factor mutant, *puf6Δ*, which we identified in an unrelated study as having reduced Ty1 cDNA levels. The *puf6Δ* mutant was not found in this screen because *med1Δ puf6Δ* progeny were not viable, but *rtt101Δ puf6Δ* progeny had no retrotransposition events. The average Ty1 cDNA level in two biological replicates of the *puf6Δ* single mutant was 18% of that in a congenic wild-type strain. To confirm that these seven ribosome biogenesis factor genes are required for efficient retrotransposition, each was deleted in strain JC3807, which harbors a chromosomal Ty1*his3AI* element. The *dbp7Δ* mutant had the strongest retrotransposition defect (Figure 4A), consistent with the low levels of Ty1 cDNA in this mutant (Table 2). Retrotransposition was reduced >10-fold in the *hcr1Δ*, *mrt4Δ*, and *puf6Δ* mutants and approximately 4-fold in *bud21Δ* and *loc1Δ* mutants. Deletion of the seventh ribosome biogenesis factor gene, *RKM4* resulted in very slow growth, and the frequency of retrotransposition in four independent isolates varied more than 10-fold (data not shown). Consequently, the *rkm4Δ* mutant was not analyzed further.

To determine whether these six *rhfΔ* mutants with reduced retrotransposition and cDNA levels have a defect in translation of Ty1 RNA, we compared Ty1 RNA and Gag levels in the mutants to those in the wild-type strain. The amount of Ty1 RNA relative to *PYK1* RNA in each strain was determined by Northern blot analysis (Figure 4B). Ty1 RNA levels in each mutant were equivalent or increased relative to the wild-type strain, and only the full-length Ty1 transcript was observed. One caveat of this analysis, however, is that the stability of *PYK1*mRNA could be altered in ribosome biogenesis mutants because of translation defects, resulting in changes in the Ty1/*PYK1* RNA ratio that do not result solely from altered Ty1 RNA levels. Therefore, quantitative real-time RT-PCR (qRT-PCR) was performed to measure the level of Ty1 RNA relative to the nuclear non-coding *SNR6* RNA (Figure 4C, left panel). Ty1 RNA levels, as measured by qRT-PCR, were not decreased in the *bud21Δ*, *dbp7Δ*, *hcr1Δ*, *loc1Δ*, *mrt4Δ*, or *puf6Δ* mutant, demonstrating that the retrotransposition defects in these mutants are not a consequence of reduced Ty1 RNA. Moreover, this analysis revealed an 84-fold increase in Ty1 RNA in the *dbp7Δ* mutant, 3- to 33-fold increases in *bud21Δ*, *hcr1Δ*, *loc1Δ*, and *mrt4Δ* mutants and no significant change in the *puf6Δ* mutant. In contrast, an *spt3Δ* strain, which lacks a critical Ty1 transcription factor, had 14% Ty1 RNA relative to the wild-type strain. Together the data suggest that the ribosome biogenesis factors act at a post-transcriptional step in retrotransposition.

Ty1 Gag expression in the ribosome biogenesis mutants was assayed by Western blotting. As expected, both unprocessed p49-Gag and processed p45-Gag were detected in the wild-type strain (Figure 4D). The p45-Gag/p49-Gag ratio in each of the six mutants was similar to that in the wild-type strain, indicating that the efficiency of Gag processing is not affected in any of the mutants. Total Gag levels appeared to be decreased in the *bud21Δ, hcr1Δ*, *loc1Δ*, *mrt4Δ*, and *puf6Δ* mutants. To confirm this conclusion using a quantitative method, we used the chromosomal Ty1 translational reporter construct, Ty1(*gag::GFP*)-3566 in strain JC3807. The reporter consists of a chromosomal Ty1 in which the *GFP* ORF is fused to the 3′ end of *gag* at the p45-Gag processing site [35]. The p45-Gag:GFP levels were modestly reduced (44% to 81% of that in the wild-type strain) in *bud21Δ*, *hcr1Δ*, *loc1Δ*, and *puf6Δ* mutants (Figure 4E). Using qRT-PCR, we confirmed that Ty1(*gag::GFP*)-3566 RNA was not decreased in a *bud21Δ* mutant relative to the wild-type strain, so the reduction in p45-Gag:GFP to 44% is not due to Ty1(*gag::GFP*)-3566 RNA instability (Figure 4C, right panel). Taken together, these data indicate that *bud21Δ*, *hcr1Δ*, and *loc1Δ* have reduced levels of total Ty1 Gag:GFP fusion protein, despite 3- to 33-fold increases in total Ty1 RNA. In addition, the *puf6Δ* mutant has decreased Gag:GFP levels despite Ty1 RNA levels that are equivalent to the wild-type strain. Our data support the conclusion that Ty1 RNA translation or Gag protein stability is reduced in *bud21Δ*, *hcr1Δ*, *loc1Δ*, and *puf6Δ* mutants.

The p45-Gag:GFP activity was not significantly changed in the *mrt4Δ* mutant and slightly increased in the *dbp7Δ* mutant. While both these strains had significant increases in Ty1 RNA, the data do not allow us to conclude that there is a defect in Gag synthesis or stability. Further analysis will be necessary to determine whether the efficiency of Ty1 RNA translation is altered in *dbp7Δ* and *mrt4Δ* mutants.

## Discussion

The mobility of retrotransposons is tightly regulated by the host cell because of their potential as insertional mutagens and drivers of genome instability. Host-mediated repression of Ty1 mobility presents a significant barrier to identifying co-factors that are required for endogenous Ty1 element retrotransposition. Therefore, we used two independent genetic backgrounds in which endogenous Ty1 element retrotransposition is derepressed to screen for transposition-defective mutants, resulting in the identification of 275 *RHF* genes. Verification that 45 of the 275 RHFs are *bona fide* Ty1 co-factors is provided by their previous identification as co-activators of plasmid-based Ty1 or Ty3 elements. We also confirmed that six newly identified RHFs (Bud21, Dbp7, Dgf10, Hcr1, Mrt4, and Nat4) are *bona fide* Ty1 co-factors by deleting the gene that encodes them in a strain harboring a chromosomal Ty1*his3AI* element, and demonstrating that retrotransposition is significantly decreased. An additional 18 *RHF* genes were validated by a >2-fold reduction in Ty1 cDNA when each gene was deleted. Overall, one-quarter of the *RHF* genes identified here have been validated by independent approaches, suggesting that iterative SGA screening is a powerful strategy for identifying host co-factors of retrotransposition.

The SGA screen for Ty1 co-factors was not exhaustive because only 3,448 (71%) deletion strains yielded progeny that grew well enough for retrotransposition to be measured in both the *med1Δ* and *rtt101*Δ trials. Ty1 co-factor gene deletions whose phenotypes were masked by either the *rtt101Δ* or *med1Δ* mutation might also have been missed in SGA analysis. Moreover, the requirement that only those gene deletions that reduced retrotransposition ≥5-fold in four separate trials be counted may have precluded the discovery of some *bona fide* Ty1 co-factors. Indeed, deletion of several previously characterized Ty1 co-factor genes (for example, *BUD22, TEC1*, *XRN1*, *SET3*, *PAT1*, and *UPF2*) failed to reduce retrotransposition in both *rtt101Δ* trials or both *med1Δ* trials, and thus the genes were not identified as *RHF* genes. However, the stringency of the screen provides confidence that the RHFs that were identified are necessary for retrotransposition regardless of the genetic background. Although RHFs are not a comprehensive set of Ty1 co-factors, they are broadly distributed among molecular function and biological process categories, suggesting that they affect many different stages of the Ty1 replication cycle or that numerous cellular pathways influence a central process that is necessary for retrotransposition (Figure 3; see Additional file 2).

A few *RHF* genes, particularly those whose deletion results in extremely elevated Ty1 cDNA levels, may have been misidentified. This group includes *MMS2* and *CTF4*, two characterized Ty1 repressors. Moreover, we assume that *POL32*, a DNA replication and repair gene whose absence increased Ty1 cDNA more than 30-fold, is a Ty1 repressor, since many other genome maintenance genes function as Ty1 repressors [29,36]. Other genes that may have been misidentified as *RHF*s are those required for efficient splicing, because the intron within the *his3AI* indicator gene must be removed by splicing in order to be activated. However, there are only a few *RHF* genes that are known to play a role in RNA splicing (see Additional file 2).

Our study identified many *RHF* genes that are conserved in eukaryotes. More than half of the *RHF* genes have statistically robust human homologs, and multiple examples of co-factors with human orthologs were identified (Table 2). Human orthologs of *RHF* genes could play a role in retroviral replication; indeed, human orthologs of the Ty1 co-factor Dbr1 and a few repressors of Ty1 retrotransposition have been implicated in analogous roles in HIV-1 replication [37-39]. The human ortholog of *DBF20*, a novel *RHF* gene that is necessary for Ty1 cDNA accumulation (Table 2), encodes the serine-threonine kinase, NDR2. NDR2 is incorporated into HIV-1 particles and processed by the HIV-1 protease [40]; however, it has not yet been shown to influence HIV-1 replication directly. Two additional RHFs that are necessary for Ty1 cDNA synthesis or stability have human homologs that have been identified in an RNAi screen as presumptive HIV-1 co-factors: Upf3 (homolog of human UPF3B) and Snf1 (homolog of human SNF1LK) [41]. One example of an RHF that could provide a clue to facilitate the characterization of an HIV-1 co-factor is the class E vacuolar protein sorting factor, Bro1. Bro1, which was also identified previously as a Ty3 co-factor, is a homolog of ALIX, which binds to HIV-1 Gag p6 and promotes HIV-1 virion budding [42,43]. Bro1 is also a co-factor for replication of Brome Mosaic Virus (BMV), a positive strand RNA virus that replicates in *S. cerevisiae*. BMV replication takes place in membrane-bound vesicular invaginations at the perinuclear endoplasmic reticulum [44,45]. Perhaps the fact that Ty1 and Ty3 elements and BMV require Bro1 for replication rather than budding indicates that another function of Bro1 in coordinating the deubiquitinization of cargo proteins in multivesicular bodies is important for replication of all these retroelements, including HIV-1 [46].

There is significant overlap between the 275 *RHF* genes and a set of 97 genes identified in a screen for host genes that affect the replication of BMV in yeast [47]. Twenty genes were identified in both screens (P = 2.85 x 10^-5^), including 14 genes whose absence inhibited BMV replication or expression (*DHH1*, *LSM1*, *LSM6*, *UMP1*, *THP2*, *BRO1*, *MET13*, *LGE1*, *ELF1*, *SPT8*, *UFD4*, *SNF1*, *SNT1*, and *OCA4*) and six genes who absence increased BMV replication or expression (*CDC50*, *ELP2*, *SKI8*, *NUP170*, *RSC2*, and *MMS22*). This overlap could be a reflection of parallels between BMV replication complex assembly and Ty1 VLP assembly. There are notable similarities between positive-strand RNA virus replication and retroviral particle assembly, including recognition of discrete *cis*-acting signals in the RNA genome by an element-encoded protein and sequestration of the RNA in a nuclease-resistant, membrane-associated self-assembling protein core [44,45]. Therefore, the finding that Ty1 and BMV utilize an overlapping set of host co-factors may indicate that there is more similarity in the cellular processes that influence replication of positive-sense RNA viruses, retroviruses and retrotransposons than might have been expected based on the differences in their structures and mechanisms of replication.

Ribosome-associated proteins were significantly enriched among RHFs. Many features of Ty1 RNA structure and function suggest that its translation may be an important regulatory step in retrotransposition. Ty1 RNA differs from typical cellular mRNAs in that it is partitioned between translation and packaging. Moreover, the 5,700 nucleotide Ty1 RNA is an unusually long RNA in yeast, and it encodes two ORFs, the second of which is expressed only when a programmed ribosomal frameshift occurs [48]. Third, the 5′ end of Ty1 RNA, including the 53-nucleotide 5′ UTR and the first 150 nucleotides of the *gag* ORF, is predicted to form an extended stem-loop structure that is likely to play a repressive role in translation [49,50]. Thus, ribosomal proteins and ribosome biogenesis factors that function as RHFs could participate in the regulation of Ty1 RNA translation. However, our data suggest that a significant proportion of these RHFs do not influence Ty1 cDNA levels, and therefore are not likely to directly control Ty1 RNA translation. For example, deletions of genes encoding 60 S ribosomal subunit proteins Rpl33b, Rpl34a, and Rpl37a, 40 S subunit proteins Rps11a, Rps19a, and Rps27b, ribosome biogenesis factors Rsa3 and Utp30, and the ribosome-associated chaperone, Zuo1 did not reduce Ty1 cDNA levels substantially. In addition, none of the RHFs that encode mitochondrial ribosome proteins had a significant effect on Ty1 cDNA levels. Deletion of RHFs that are required for Gag expression or translational frameshifting from *gag* to *pol* would be expected to reduce the level of Ty1 cDNA, because the ratio of Gag to Gag-Pol is critical for Ty1 protein processing, and processing, in turn, is required for cDNA synthesis [51-54]. What then are the roles of ribosome-associated factors that don’t affect early steps in retrotransposition prior to cDNA synthesis? Perhaps they act indirectly by affecting gene expression or cell growth in ways that influence the localization of VLPs or the availability of cDNA for integration. Alternatively, ribosome-associated factors could act extraribosomally to influence the sub-cellular localization or fate of Ty1 RNA and associated proteins, thereby interfering with nuclear import or integration of Ty1 cDNA.

The majority of *RHF* genes, when deleted, result in ≤2-fold change in the level of Ty1 cDNA, suggesting that they exert their effects on retrotransposition at steps subsequent to the synthesis or accumulation of Ty1 cDNA. This set of RHF genes includes several chromatin organization genes that have a potential role in the integration of Ty1 cDNA into the host genome. Ty1 integrates into nucleosomes upstream of RNA polymerase III genes, but the chromatin determinants of this integration pattern are not known. A recent genome-wide analysis of Ty1 integration sites revealed a significant correlation between Ty1 integration hotspots and nucleosomes enriched for H3K14 acetylation and histone variant H2A.Z substitution [55]. *RHF* genes that act after cDNA synthesis and are known to influence chromatin organization include Snf1, Gal83, and Sip4 (components of the Snf1 complex); Caf40 and Ccr4 (components of the Ccr4-NOT core complex); Hda1 and Hda3 (components of the Hda1 deacetylase complex); Ume1 and Ume6 (components of the Rpd3L histone deacetylase complex); Ino2 and Ino4 (components of the Ino2/Ino4 transcription activator); Swr1 and Vps72 (components of the SWR1 complex, which exchanges H2A.Z for H2A in chromatin-bound nucleosomes [56,57]); and Nat4, an N(alpha)-acetyltransferase involved in the N-terminal acetylation of histone H4 and H2A [58]. These chromatin modifiers could enhance integration of Ty1 cDNA by modifying the accessibility of the target DNA. Our data indicate that Nat4 is a potent co-factor for chromosomal Ty1*his3AI* retrotransposition even though Ty1 cDNA levels are not decreased in a *nat4Δ* mutant. Thus, Nat4 may modulate Ty1 retrotransposition through its effects on the chromatin structure of the target DNA. This finding may be useful in understanding the role of Nat4 in chromatin dynamics, which is poorly understood.

Deletion of 43 *RHF* genes resulted in ≥ 2-fold decrease in endogenous Ty1 cDNA levels (Table 2). A retrotransposition defect has previously been reported for eight of the 43 corresponding *rhfΔ* mutants, and we verified the retrotransposition defect in seven additional *rhfΔ* single mutants. Thus, the reduced cDNA levels in these mutants provide independent verification that these 43 RHFs affect Ty1 elements globally, rather than having specific effects on the marked Ty1*his3AI* element. This class includes three genes of unknown function: *DGR2*, *HIT1*, and *OCA4*. A forth gene, *YDL124W*, encodes an evolutionarily conserved NADPH-dependent alpha-ketoamide reductase, but its cellular function has not been elucidated. However, most of these *RHF* genes encode proteins that are involved in RNA metabolism, raising the possibility that they affect the metabolism of Ty1 RNA or its tRNA^iMet^ primer or trafficking of Ty1 RNA between different functions in the mobility cycle. Almost one-third of the RHFs that are required for efficient cDNA accumulation are ribosome-associated. While these RHFs could act indirectly or extraribosomally, at least a few may influence the translation of Ty1 RNA. These include ribosome biogenesis factors, Bud21, Hcr1, Loc1, and Puf6, whose absence resulted in decreased Ty1 Gag:GFP fusion protein levels despite wild-type or increased levels of Ty1 RNA (Figure 4).

The RHF Bud21, also known as Utp16, is a component of the small ribosomal subunit processosome that contains U3 snoRNA. The level of the 40 S subunit is markedly decreased in a *bud21Δ* mutant [59]. Hcr1 encodes eIF3j, a dual function protein involved in translation initiation as a component of translation initiation factor 3 and in processing of 20 S pre-rRNA, a precursor of the 40 S subunit. When *BUD21*or *HCR1* is deleted, Gag:GFP fusion protein levels are reduced to 44 and 52% of the wild-type level, respectively (Figure 4E); however, Ty1 RNA levels are increased 11-fold and 3-fold, respectively (Figure 4C). Thus, Ty1 RNA translation may be very sensitive to mutations that perturb 40 S ribosomal subunit formation because of stable secondary structure within the 5′ UTR. Another ribosome biogenesis mutant with reduced 40 S subunit formation, *bud22Δ*, also has a reduced level of Ty1 Gag protein; however, Ty1 RNA is not increased in *bud22Δ* mutants [14]. Moreover, the ratio of p45-Gag to p49-Gag is significantly decreased in a *bud22Δ* mutant, but we did not observe an obvious Gag processing defect in the *bud21Δ* or *hcr1Δ* mutant. Thus, the mechanism by which *BUD21* and *HCR1* affect Ty1 RNA translation is likely to be different from that of *BUD22*. The simplest interpretation of our findings is that Bud21 and Hcr1 are necessary for efficient of Ty1 RNA translation via their roles in ribosome biogenesis, although other models, including indirect effects on Gag synthesis or stability are also consistent with our data.

The RHFs Puf6 and Loc1 are required for biogenesis of the 60 S ribosomal subunit. Interestingly, both also bind *ASH1* mRNA and mediate its translational repression and localization to the bud tip [60]. Another RHF that is required for Ty1 cDNA accumulation, YDL124W, also binds to *ASH1* RNA [61]. In contrast to *ASH1* mRNA, Ty1 RNA translation may be reduced in *puf6Δ* and *loc1Δ* mutants. Moreover, Ty1 mRNA is not localized to the bud tip like *ASH1* mRNA, but it is localized to microscopically distinct cytoplasmic foci known as T bodies or retrosomes [62,63]. It is possible that Puf6 and Loc1 promote translation of Ty1 RNA simply via their effects on biogenesis of the 60 S subunit. However, Loc1 and Puf6 have been implicated in the localization of specific ribosomal protein paralogs and the formation of ‘specialized’ ribosomes that are required for the regulated translation of *ASH1* mRNA [64]. Based on this model, it is also conceivable that Loc1 and Puf6 are involved in the formation of ribosomes containing specific ribosomal paralogs that are necessary for the regulated translation of Ty1 RNA. A third possibility is that Loc1 and Puf6 bind Ty1 RNA directly and influence its translation or localization in the cell.

In contrast to the other ribosome biogenesis factors that we analyzed, Ty1 Gag-GFP levels were not decreased in the *dbp7Δ* and *mrt4Δ* mutants (Figure 4E), but Ty1 RNA is elevated > 80-fold and >30-fold, respectively (Figure 4C). Thus, the translational efficiency of Ty1 RNA could be reduced in these mutants. Dbp7 is a putative ATP-dependent RNA helicase required for formation of mature 25 S rRNA, an RNA component of 60 S ribosomal subunits. Mrt4 is a paralog of RPP0, which encodes P0, an rRNA binding component of the ribosomal stalk. The *RPP1A* gene, which encodes a second ribosomal stalk protein, P1, was also identified here and in a previous study as a Ty1 co-factor (Table 1). The ribosomal stalk plays an essential role in recruiting translation factors, and P0 interacts with the ribosomal translocation factor, eEF-2 [65]. Mrt4 is bound to pre-ribosomal particles in the nucleus and is exchanged for P0 in the cytoplasm [66-68]. Amino-acid substitutions in the essential *RPP0* gene block Ty1 retrotransposition, reportedly because of effects on programmed ribosomal frameshifting [69]. Thus it is reasonable to hypothesize that *mrt4Δ* has reduced Ty1 transposition and cDNA levels because P0 association with cytoplasmic ribosomes is partially defective in the absence of Mrt4. However, we do not observe any defects in proteolytic processing in *mrt4Δ* mutants, which is not consistent with a defect in Ty1 frameshifting. Thus, further investigation is needed to understand the defect in retrotransposition in *dpb7Δ* and *mrt4Δ* mutants.

## Conclusions

Iterative synthetic genetic array analysis is a powerful tool to identify genes that are required for complex phenotypic traits influenced by multiple cellular pathways. We used this strategy to identify 275 presumptive co-factors of Ty1 retrotransposon mobility, one-quarter of which were validated by independent approaches. Ty1 co-factors participate in numerous cellular pathways and include those that affect the accumulation of Ty1 cDNA and those that act at later stages in retrotransposition. Our results highlight the extensive reliance of Ty1 on host co-factors in the mobility cycle. A significant number of Ty1 co-factors are ribosome-associated, suggesting that translational regulation plays a central role in coordinating different steps in Ty1 retrotransposition.

Many Ty1 co-factors have statistically significant human homologs, underscoring the role of conserved eucaryotic cellular pathways in Ty1 retrotransposition. Screens for human genes that are required for HIV-1 replication have uncovered over 1,000 potential co-factors; however, only a relatively small fraction of these co-factors have been validated [70]. Identification of Ty1 co-factor genes that are conserved from yeast to humans can lead to the validation and characterization of human effectors of steps in retrovirus replication that are shared among LTR-retrotransposons and retroviruses and therefore likely to be essential steps in retroelement replication.

## Methods

### Media

Standard yeast media were used [71], except when synthetic complete (SC) medium was supplemented with G418, in which case 0.1% monosodium glutamate was used in place of ammonium sulfate. SC medium containing monosodium glutamate is referred to as SC[msg].

### Construction of SGA query strains

The genotype of strains used in this study, all of which are derivatives of congenic strains BY4741 and BY4742, are described in Table 3. Oligonucleotide primers used in PCR-mediated gene disruption are provided in Table 4. The yeast ORF deletion collection in strain BY4741 was obtained from Research Genetics Inc. (renamed Invitrogen MapPairs, catalog no. 95401.H2P). Strain Y9230 [31] was a gift of Dr. Charles Boone. Strain JC4436 is an *rtt101Δ::LEU2* derivative of Y9230. The *rtt101Δ::LEU2* allele was PCR-amplified using primers Rtt101K5 and Rtt101K3 and pRS405 DNA as a template and transformed into strain Y9230. In strains JC4501 and JC4502, the 3′ UTR of *YJRWTy1-2* was marked with *his3AI*, and *MET15* was inserted between *YJRWTy1-2* and *YJR030C* by one-step PCR-mediated gene disruption. PCR SOEing [72] was used to synthesize a DNA fragment containing the 3′ end of Ty1*his3AI-Δ1*[28], the *MET15* gene, and genomic DNA sequences downstream of *YJRWTy1-2*. To accomplish this, we synthesized two PCR products, one using TYBOUT2 and Ty1JR2-2 L as primers and plasmid pGTy1*his3AI-[Δ1]* DNA as a template, the other using Ty1JR2-3 L and Ty1JR2-4 as primers and pRS401 DNA as a template. The two fragments were then annealed and amplified by PCR using primers TYBOUT2 and Ty1JR2-4. The resulting 3 kb fragment was inserted into the vector, pCR2.1-TOPO using the Invitrogen TOPO TA Cloning kit (Invitrogen, Carlsbad, CA, USA). The plasmid insert was verified by restriction-site mapping and sequencing. The plasmid insert was amplified using primers TYBOUT2 and TY1JR2-4 and the resulting DNA fragment was transformed into strains Y9230 and JC4436 by one-step gene disruption to yield strains JC4501 and JC4502, respectively. The *med1Δ:LEU2* allele in strain JC4808 was constructed by PCR using primers PJ71 and PJ72 and pRS405 as template DNA. The resulting PCR product was transformed into JC4501 to yield JC4808.

**Table 3:** Strain names and genotypes

**Strain**	**Genotype**	**Source**
BY4741	*MAT*a*, his3Δ1, leu2Δ0, ura3Δ0, met15Δ0*	[73]
BY4742	*MAT*α*, his3Δ1, leu2Δ0,ura3Δ0, lys2Δ0*	
Y9230	*MAT*α*, can1Δ:: STE2pr-URA3, lyp1Δ1, ura3Δ0, leu2Δ0, his3*Δ*1, met15Δ0*	[31]
JC3807	*MAT*a, *met15Δ0*, *his3Δ1*, *leu2Δ0*, *ura3Δ0,*Ty1*his3AI-*3114, Ty1*(gag::GFP*)*-*3566	[35]
JC4436	*MAT*α*, can1Δ::STE2pr-URA3, lyp1Δ1, ura3Δ0, leu2Δ0, his3*Δ*1, met15Δ0 rtt101Δ::LEU2*	This study
JC4501	*MAT*α*, can1Δ::STE2pr-URA3, lyp1Δ1, ura3Δ0, leu2Δ0, his3*Δ*1, met15Δ0, YJRWTy1-2-his3AI-MET15*	This study
JC4502	*MAT*α*, can1Δ::STE2pr-URA3, lyp1Δ1, ura3Δ0, leu2Δ0, his3*Δ*1, met15Δ0, YJRWTy1-2-his3AI-MET15, rtt101Δ::LEU2*	This study
JC4808	*MAT*α*, can1Δ::STE2pr-URA3, lyp1Δ1, ura3Δ0, leu2Δ0, his3*Δ*1, met15Δ0, YJRWTy1-2-his3AI-MET15, med1Δ::LEU2*	This study
JC5221	*MAT*a *met15Δ0 his3Δ1, leu2Δ0, ura3Δ0,*Ty1*his3AI-*3114,Ty1*(gag::GFP*)*-*3566, *puf6Δ::kanMX*	This study
JC5256	*MAT*a, *met15Δ0, his3Δ1, leu2Δ0, ura3Δ0,* Ty1*his3AI-*3114, Ty1*(gag::GFP*)*-*3566, *loc1Δ::kanMX*	This study
JC5379	*MAT*a, *met15Δ0, his3Δ1, leu2Δ0, ura3Δ0,*Ty1*his3AI-*3114, Ty1*(gag::GFP*)*-*3566,*dbp7Δ::kanMX*	This study
JC5391	*MAT*a, *met15Δ0 his3Δ1, leu2Δ0, ura3Δ0,*Ty1*his3AI-*3114, Ty1*(gag::GFP*)*-*3566,*bud21Δ::kanMX*	This study
JC5392	*MAT*a, *met15Δ0 his3Δ1, leu2Δ0, ura3Δ0,*Ty1*his3AI-*3114, Ty1*(gag::GFP*)*-*3566,*hcr1Δ::kanMX*	This study
JC5394	*MAT*a, *met15Δ0 his3Δ1, leu2Δ0, ura3Δ0,*Ty1*his3AI-*3114, Ty1*(gag::GFP*)*-*3566, *mrt4Δ::kanMX*	This study

**Table 4:** Oligonucleotide primers used in this study

**Primer**	**Sequence**
Ty1JR2-4	CTTCTGTTATCTTCTGTTAAAGTAAGGCAACTGAGAAATATGTGACTGTGCGGTATTTCACACCG
Ty1JR2-2 L	GTGCACTCTCAGTACAATCTGCAACAAATTGATAAGCAATGC
Ty1JR2-3 L	GCATTGCTTATCAATTTGTTGCAGATTGTACTGAGAGTGCAC
TYBOUT2	GTGATGACAAAACCTCCTCCG
TY5253A	GGACAGATTCACTTATCGCGTGT
Rtt101K3	CTATCTCAGTAGTTAGGTAATATATAAGATGGCACCAGTCCTGTGCGG TATTTCACACCG
Rtt101K5	TTTTTACTGGTATAAATTCTCGTA CGG GTT CAC AGG AAC AAG ATT GTA CTG AGA GTG CAC
PJ71	AAAAGCCAACAAAACTCTTTTGGAGATGGTAGATTGTACTGAGAGTGCAC
PJ72	GAGTGTACGGTCCACAATGTGTATTTGAGCCACTCCGTACCTCCTCTGTGCGGTATTTCACACCG
PJ748	GCTTCGTATGGCAACCAACC
PJ750	TTCGCGAAGTAACCCTTCGTGGA
PJ751	GTAAAACGGTTCATCCTTATGCAG
PJ913	AGAAGAATGATTCTCGCAGC
PJ914	CCAGCTTTTGTTCCC

Strains JC5221, JC5256, JC5379, JC5391, JC5392, and JC5394 were constructed by amplifying the appropriate *orfΔ:kanMX* allele from the *MAT*a deletion collection and transforming strain JC3807 with the PCR product. All strains constructed by PCR-mediated gene disruption were checked for precise replacement of the wild-type allele by the PCR fragment using at least two diagnostic PCR reactions: one with a set of primers that flank the ORF and another with a flanking primer and a primer that hybridizes to *kanMX* sequences.

### Modified SGA analysis

We used a modification of the SGA protocol of Tong and Boone [31] to accommodate a liquid medium platform and a semi-quantitative assay of Ty1*his3AI* retrotransposition in each viable haploid strain. Trials 1 and 2 (using strain JC4502 as a query) were performed with a Thermo Scientific Matrix Hydra DT liquid handling robot. Trials 3 and 4 (using JC4808 as a query) were performed using a Beckman Coulter Biomek FX liquid handling robot.

Using a slot-pin replicator, yeast ORF deletions strains were inoculated into 96-well plates containing 200 μL YPD broth with 200 μg/mL G418 in each well. Plates were incubated at 30° for 2 days. The query strain (JC4502 or JC4808) was grown in YEPD broth at 30° overnight. Strains were mated by transferring 5 μL of each ORF deletion strain and 5 μL of the query strain into 200 μL YPD broth and incubating at 30° for 3 days. To select diploids, 5 μL of each mating mixture was transferred to 200 μL SC[msg]-Met-Leu + 200 μg/mL G418 broth, and cultures were incubated at 30° for 3 days. Cultures of diploid strains (5 μL) were transferred into 200 μL sporulation medium + His + Ura and incubated for 14 days at 24°. Duplicate 5 μL aliquots of each spore culture were transferred into 200 μL SC[msg]-Ura-Arg + 60 mg/L canavanine broth, and cultures were incubated at 30° for 5 days. Subsequently, 5 μL of each culture was transferred to 200 μL SC[msg]-Ura-Arg-Met-Leu + 60 mg/L canavanine + 200 μg/mL G418, and cultures were incubated at 30° for 5 days. A 5 μL aliquot of each culture was transferred into 200 μL of YPD + 200 μg/mL G418 broth and incubated at 20° for 5 days. In one trial with strain JC4502 and one trial with strain JC4808, the appropriate parental query strain was added to an empty well in each plate at the same dilution (that is, 5 μL of an overnight culture in 200 μL of YPD + 200 μg/mL G418 broth). Finally, 10 μl of each culture was spotted by hand onto YPD + 200 μg/ml G418 agar and onto SC-His agar (query strain JC4502), or 20 μL of each culture was spotted robotically (query strain JC4808), and all plates were incubated at 30° for 4 days. Duplicate plates were assigned to Trial 1 or Trail 2 (JC4502 query strain) or Trial 3 or Trial 4 (JC4808 query strain). Growth on YPD + G418 was evaluated and recorded, and retrotransposition was evaluated by individually counting His^+^ papillae at each address on SC-His agar. Results were tracked using an MS Excel spreadsheet and an MS Access database.

To determine the probability that RHFs identified by screening with one query strain would also be identified in the other screen with a second query strain, we calculated the hypergeometric distribution ( http://www.alewand.de/statlab/tabdiske.htm). The list of 275 candidate RHF genes was submitted to FunSpec ( http://funspec.med.utoronto.ca), and the statistical significance of values for enrichment in MIPS functional categories were obtained using the Bonferroni correction.

### cDNA analysis

The level of unintegrated Ty1 cDNA relative to genomic Ty1 element DNA was determined by the method of Lee *et al.*. [34], with minor alterations. Independent colonies of each strain were inoculated into 10 mL YPD broth, and each culture was incubated at 20° for 2 days. Genomic DNA prepared from each culture was digested with SphI. Ty1 cDNA was detected by Southern blot analysis using a ^32^P-labeled *TYB1* riboprobe. The Ty1 cDNA band was quantified relative to two genomic Ty1 bands by phosphorimaging, as described previously [28].

### Northern blot analysis

Total RNA was prepared from cells grown to mid-log phase at 20°, denaturated by the addition of glyoxal, separated on a 1% agarose gel and transferred to a Hybond N membrane (Amersham) as described previously [74]. Plasmids pGEM-TYA1 and pGEM-PYK1 [75] were used as DNA templates for riboprobe synthesis. Bands were quantified by phosphorimaging.

### Western blot analysis

Strains were grown in YEPD broth at 20°C to mid-logphase and four A600 units of cells were pelleted. Proteins were extracted from the cell pellet by the addition of 200 μL of lysis buffer (20 mM Tris–HCl pH 7.5, 150 mM NaCl, 1.8 mM MgCl_2_, 0.5% IGEPAL CA-630 (Sigma-Aldrich), cOmplete Mini EDTA-free protease inhibitor (Roche), 1 mM DTT, 80 U/mL RNasin (Promega)) and 200 μL of acid-washed beads followed by vortexing for 4X 3 min with a 3-min incubation on ice between each vortexing. A 45-μL aliquot of the supernatant was removed to which 5 μL of 5X SDS-PAGE loading buffer was added. The samples were incubated at 70°C for 10 min and 6 μL of the supernatant was separated on a 10% SDS-PAGE gel. The proteins were transferred to a PVDF membrane and the membrane was incubated in 5% non-fat dry milk and 1X PBST for 1 h, followed by a 1-h incubation with affinity-purified anti-Gag polyclonal antibody diluted 1:2,000 in 1% non-fat dry milk in 1XPBST or anti-Tubulin polyclonal antibody (Chemicon International) diluted 1:10,000 in 2.5% non-fat dry milk in 1XPBST as a loading control. Subsequently, the membrane was incubated with HRP-conjugated secondary antibodies and SuperSignal® West Pico Chemiluminescent Substrate (Pierce) and exposed to film.

### Retromobility frequency assays

The frequency of Ty1*his3AI* retrotransposition in strains JC3807, JC5221, JC5256, JC5379, JC5391, JC5392, and JC5394 was determined by inoculating YPD broth with a single colony of each strain. The cultures were grown to saturation at 30°, diluted 1:1,000 in YPD broth and incubated at 20° until saturation (6 days for the *dbp7Δ* derivative of JC3807; 3 days for all other strains). A 1:1,000 dilution of a 1 μL aliquot of each strain was plated on YPD agar to determine the titer of the culture. One millileter aliquots of the remaining culture were plated on SC-His agar. All plates were incubated at 30° for 3 days, and the number of colonies on each plate was counted. The retromobility frequency is the number of His^+^ colonies divided by the total number of cells plated on SC-His agar. The average frequency and standard error for each strain were calculated from nine to fifteen cultures.

### Quantitative real-time PCR

Three independent yeast colonies of each strain were grown overnight in YPD broth at 30. Cultures were diluted 1:25 in YPD and incubated at 20° for 3 h. Cells were pelleted, washed in ice-cold water, pelleted again and frozen on dry ice. Cell pellets were thawed on ice, and RNA was extracted with the MasterPure Yeast RNA Purification Kit (Epicentre) according to the manufacturer’s instructions. DNA was removed from approximately 10 μg of nucleic acid from each preparation using TURBO DNA-free (Invitrogen) according to the manufacturer’s instructions.

Equivalent amounts of RNA (approximately 1 μg) were used to generate negative-strand cDNA with the First-Strand cDNA Synthesis Kit for Real-Time PCR (USB) according to the manufacturer’s instructions; controls lacking reverse transcriptase (RT) were run in parallel. qPCR was performed using HotStart-IT SYBR Green qPCR 2X Master Mix (USB). Each cDNA sample was analyzed using primers TY5253A and PJ748 to detect Ty1 RNA or primers PJ913 and PJ914 to detect Ty1(*gag:: GFP*) RNA. As a normalization control, each cDNA sample was also analyzed using primers JC750 and JC751 to detect *SNR6* RNA. Triplicate qPCR reactions were performed using each primer set. A Ct value for each reaction was determined by the Applied Biosystems 7500 Fast Real-Time PCR System software using the Manual Ct and Manual Baseline. The data were accepted if the pairwise differences in Ct among three replicates was <0.5, and if the difference between averaged + RT samples and -RT controls was >5.0 Ct. For each Ty1 or Ty1(*gag::GFP*) primer set, the Ct of triplicate reactions were averaged to generate Ct_Ty1_. For the *SNR6* primer set, the Ct of triplicate reactions was averaged to generate Ct_*SNR6*_.

The Ct_Ty1_ and the Ct_*SNR6*_ were determined for three independent RNA samples from each strain. To correct for the inherent differences arising from analyzing biological replicates, the Ct_Ty1_ and the Ct_*SNR6*_of each biological replicate was averaged to generate the Ct_Total_, and then the median Ct_Total_of three biological replicates was subtracted from each Ct_Total_to generate a correction factor for each biological replicate. This correction factor was either added to or subtracted from the Ct_Ty1_ and Ct_*SNR6*_ofeach biological replicate, depending on whether the Ct_Ty1_ or Ct_*SNR6*_ was lesser or greater than the Ct_Total_, respectively. The fold-change in Ty1 RNA level between one wild-type RNA sample and one mutant RNA sample analyzed in a single experiment was calculated using the following equation [76], in which E corresponds to the average amplification efficiency of each strain, and Ct refers to the corrected Ct_Ty1_ and the Ct_*SNR6*_for each biological replicate of each strain:

$$\begin{array}{l} Fold-change\;in\;Ty1\;RNA=\left[E_{TY1}{}^{\left(WT\;Ct_{Ty1}{{}^{-rhf\Delta \;Ct}}_{Ty1}\right)}\right]\\ \;/\left[E_{SNR6}{}^{\left(WTCT_{SNR6}{{}^{-rhf\Delta CT}}_{SNR6}\right)}\right] \end{array}$$

In the case of the wild-type strain, the fold-change was 1.0. The mean of the fold-change in the Ty1 RNA in each mutant strain relative to the wild-type strain in three sets of biological replicates of each strain was determined, and the standard error of the mean was calculated.

## Competing interests

The authors declare that they have no competing interests.

## Authors’ contributions

JR participated in the design of the experiment and carried out the SGA screens and constructed the databases. AK and EG carried out the assay for Ty1 cDNA levels in *rhfΔ* mutants. RJP made strains lacking ribosome biogenesis mutants and carried out the molecular genetic assays on these mutants. MJC conceived the study, participated in the design and wrote the manuscript. All authors read and approved the manuscript.

## Supplementary Material

Additional file 1 **Table of *RHF * genes, human homologs and Ty1 cDNA levels in *rhfΔ *mutants.** Click here for file

Additional file 2 **Tables of GO function and GO process categories of *RHF * genes.** Click here for file
